# Fast negative breakdown in thunderstorms

**DOI:** 10.1038/s41467-019-09621-z

**Published:** 2019-04-09

**Authors:** Julia N. Tilles, Ningyu Liu, Mark A. Stanley, Paul R. Krehbiel, William Rison, Michael G. Stock, Joseph R. Dwyer, Robert Brown, Jennifer Wilson

**Affiliations:** 1 Space Science Center, Department of Physics, University of New Hampshire, Durham, NH 03824 USA; 2 Langmuir Laboratory for Atmospheric Research, New Mexico Tech, Socorro, NM 87801 USA; 3 Earth Networks, Germantown, MD 20876 USA; 4 NASA, John F. Kennedy Space Center, FL 32899 USA

## Abstract

Thunderstorms are natural laboratories for studying electrical discharges in air, where the vast temporal, spatial, and energy scales available can spawn surprising phenomena that reveal deficiencies in our understanding of dielectric breakdown. Recent discoveries, such as sprites, jets, terrestrial gamma ray flashes, and fast positive breakdown, highlight the diversity of complex phenomena that thunderstorms can produce, and point to the possibility for electrical breakdown/discharge mechanisms beyond dielectric breakdown theory based mainly on laboratory experiments. Here we present one such confounding discovery, termed fast negative breakdown, that does not fit with our current understanding of dielectric breakdown. Our adaptation of radio astronomy imaging techniques to study extremely transient lightning-associated events confirms that electrical breakdown in thunderstorms can begin with oppositely-directed fast breakdown of negative polarity, similar and in addition to fast positive breakdown expected from conventional dielectric theory and recent observations. The discovery of fast negative breakdown calls for an addendum to the physical description of electrical discharges in air.

## Abstract (web summary)

Recent studies have shown that lightning is initiated by a newly-recognized discharge process called fast positive breakdown. Here, the authors present observational evidence of fast breakdown but of negative polarity, seemingly contrary to current understanding of discharge physics.

## Introduction

Despite over 250 years of dedicated research^1^, the details of lightning initiation remain unknown. However, compact intra-cloud discharges called narrow bipolar events (NBEs)^2–4^ almost always occur either in isolation or at the beginning of a lightning flash^3–8^, strongly suggesting that they are the initiating breakdown events of thunderstorm electrical discharges. NBEs are also of particular interest for thunderstorm research and radio science, because they are the most powerful natural emitter of very high frequency (VHF) radio waves on Earth^3^. Rison et al.^8^ recently discovered that NBEs are generated by a newly recognized process, termed fast positive breakdown, and concluded that many or possibly all lightning flashes are initiated by fast positive breakdown. This finding is consistent with a lightning initiation theory proposed several decades ago^9–11^, which hypothesized that lightning is initiated by a succession of positive streamers, resolving the conundrum that measured thunderstorm electric fields are well below the dielectric breakdown threshold^12–15^. The results indicate that the fast breakdown originates in localized regions of relatively strong electric field^12^, with the positive streamers being initiated by ice hydrometeors at field strengths well below the dielectric breakdown threshold of air. Streamers^16,17^ are cold, filamentary plasma discharge waves, and once initiated they are believed to propagate in the thunderstorm electric field, eventually leading to the development of the first lightning channel, known as a lightning leader^18,19^.

Laboratory^20,21^ and modeling^22–24^ work have indeed found that positive streamers, which propagate in the direction of the electric field and carry electric currents in the propagation direction, can be initiated from isolated ice hydrometeors placed in an electric field well below the threshold for air electrical breakdown. Moreover, it has been found that negative streamers, which propagate in the opposite direction, are absent during streamer initiation from the hydrometeors. The fast positive breakdown reported by Rison et al.^8^ carries a current in its propagation direction, though with much larger current magnitude (~50 kA), spatial extent (~500 m), and temporal scale (~10 μs) than for a single streamer (~1 A, ~1 m, and ~0.1 μs, respectively, assuming an altitude of 9 km, or pressure of about 30 kPa)^17,22–24^. Therefore, a natural explanation for fast positive breakdown is that it is composed of a system of positive streamers or a discharge wave driven by positive streamers^8^. The conclusion thus drawn from the last few decades of culminating research is that electrical breakdown in thunderstorms starts with a positive polarity discharge.

**Fig. 1:**
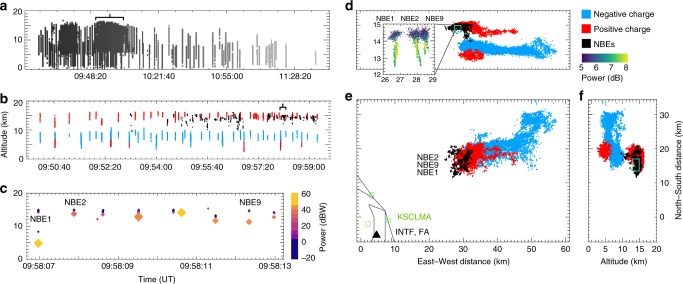
Lightning mapping observations of the 24 August 2016 storm by the Kennedy Space Center Lightning Mapping Array. **a** Overview of the height-time evolution of the 3-h storm, **b** 10 min of enhanced activity during the bracketed interval of **a**, colored by the lightning-inferred polarity of the storm charge (red = upper and lower positive charge, blue = mid-level negative charge), showing the NBE events (black), **c** zoomed-in view of 6 s of high-rate NBE activity (~40 NBEs within 1 min), indicated by the bracket in **b**, colored and sized by VHF source power and showing NBEs 1, 2, and 9. Note that the initial high-power sources of the NBEs (large diamond markers) were mis-located in altitude from the subsequent, smaller, and correctly located lower-power sources (blue markers), with NBE 1 altitude being particularly incorrect (see Methods). The low-power sources indicate that all 10 NBEs occurred at similar altitudes between 13 and 15 km MSL, immediately below or within the inferred upper positive charge region. **d**–**f** Plan and vertical cross-section views of the storm charge structure, colored by charge as in **b** and showing that the NBEs occurred in close proximity to a positively charged western anvil of the normally electrified storm. The inset in the east–west cross-section of **d** shows the full-duration INTF centroid observations for each of the three NBEs relative to the storm location, with marker colors corresponding to relative VHF power. The inset shows that each NBE had a vertical extent of ≃1.5 km on the lower edge of the upper positive charge region. The plan view of **e** shows the NBE locations as white circles. The cyan boxes in **d** and **f** indicate their locations in the vertical cross-sections. The black triangle in **e** indicates the INTF location at KSC

Here we report observations of electrical breakdown in thunderstorms that starts with negative polarity discharges. In 2016 and 2017, as a follow-up study, we deployed an improved version of the broadband VHF radio interferometer used by Rison et al.^8^ to Kennedy Space Center in Florida. The Florida storms were found to be prolific generators^25^ of high-power 30–50 dBW (1–100 kW) NBEs, some of which were caused by fast breakdown that propagated in the opposite direction of similarly located fast positive events, which were downward-directed. In both cases, and in a number of different storms, the discharges in question occurred between the mid-level negative and upper positive charge regions of the storms (e.g., Fig. 1), typical of intra-cloud lightning. Thus, upward development would be indicative of negative-polarity breakdown. Because this conflicts with the prevailing view that lightning starts with positive breakdown, and because the non-imaging centroid technique used in previous studies^8^ cannot rule out the possibility that the propagation is an apparent effect due to a succession of downward positive events starting retrogressively at higher altitudes, we present a combination of radio imaging and centroid analyses to confirm that fast negative breakdown is dominated by a propagating localized source, thereby showing that breakdown can begin with negative as well as positive polarity. This finding challenges our understanding of dielectric breakdown of air.

## Results

### Fast breakdown events in an NBE-prolific thunderstorm

The observations were obtained at Kennedy Space Center, Florida, from a 24 August 2016 thunderstorm located offshore. The observations include three-dimensional lightning mapping array (LMA) data^5,26^ from the 10-station Kennedy Space Center Lightning Mapping Array (KSCLMA), VHF waveforms recorded by the New Mexico Tech Broadband Interferometer^27^ (INTF), and waveforms recorded by a flat-plate Fast Antenna (FA) that measured the change in vertical electric field at the ground with a 100-μs decay constant.

Figure 1 shows an overview of the LMA observations for the 24 August storm and the events of interest. Figure 1a shows the full duration of the source altitudes vs. universal time (UT), with LMA sources colored according to time. A 10-min period of the storm (black bracket in Fig. 1a) is expanded in Fig. 1b, d–f, where the LMA sources are colored according to the polarity of the lightning discharge events, with red sources indicating positive storm charge, and blue sources indicating negative storm charge^28–30^. High-power NBEs are colored in black, and typically occurred below the upper positive charge region on the upwind side of the storm. This 10-min period is marked by an increase in flash rate (from about 1 to 5 fully developed flashes per minute) and flash initiation altitude (from about 8–10 to 10–15 km above mean sea level (MSL)), as well as an increase in isolated NBE occurrence (from about 1 to 15 min^−1^) at the flash initiation altitudes. Such high NBE initiating altitudes are not unusual for Florida storms^6,31^, but the NBE occurrence rate is exceptional and is in stark contrast to previous studies^32^.

**Fig. 2:**
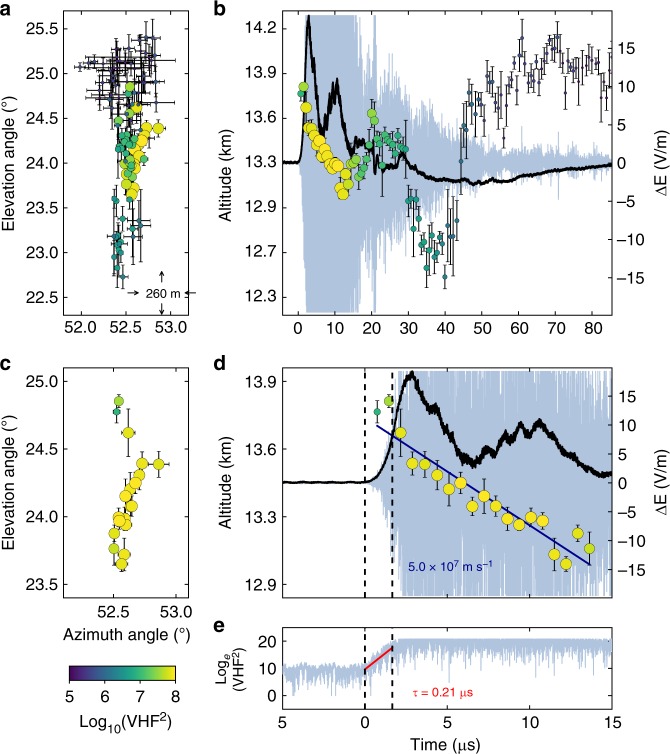
Interferometer data for NBE 1. **a**, **c** Radiation centroids (circular markers, colored and sized by VHF power) for NBE 1, plotted in elevation vs. azimuth, showing the breakdown activity was primarily vertical. Each marker denotes the average angular position of the 128 source solutions in each 0.7-μs window, and error bars denote the standard deviations. **b**, **d** Radiation centroids and fast electric field change (sferic) observations (black waveform) superimposed on the VHF waveform (gray), showing the downward propagation of the VHF source. The positive sferic waveform is indicative of a downward-directed current, consistent with the NBE occurring below the storm’s upper positive charge, and indicating the downward development was due to positive-polarity breakdown. The breakdown descended ≃600 m in 13 μs, corresponding to a speed of ≃5 × 10^7^ m s^−1^. **e** Semilog plot of the VHF power vs. time, showing the fast exponential rise of the radiation (rise time *τ* = 0.21 μs), coincident with the fast rise of the electric field change (**d**). The peak current of the breakdown was −75 kA

**Fig. 3:**
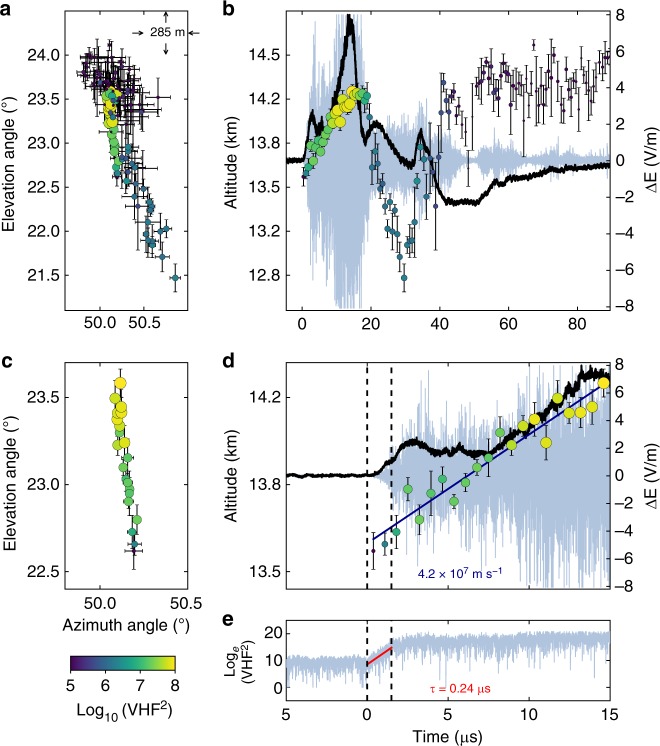
Interferometer data for NBE 2. **a**, **c** Radiation centroids (circular markers, colored and sized by VHF power) for NBE 2, plotted in elevation vs. azimuth, showing the breakdown activity was primarily vertical. Each marker denotes the average angular position of the 128 source solutions in each 0.7-μs window, and error bars denote the standard deviations. **b**, **d** Radiation centroids and fast electric field change (sferic) observations (black waveform) superimposed on the VHF waveform (gray), showing the upward propagation of the VHF source. The positive sferic waveform is indicative of a downward-directed current, consistent with the NBE occurring below the storm’s upper positive charge, and indicating the upward development was due to negative-polarity breakdown. The breakdown ascended ≃600 m in 15 μs, corresponding to a speed of ≃4 × 10^7^ m s^−1^. **e** Semilog plot of the VHF power vs. time, showing the fast exponential rise of the radiation (rise time *τ* = 0.24 μs), coincident with the fast rise of the electric field change (**d**). The peak current of the breakdown was −47 kA

**Fig. 4:**
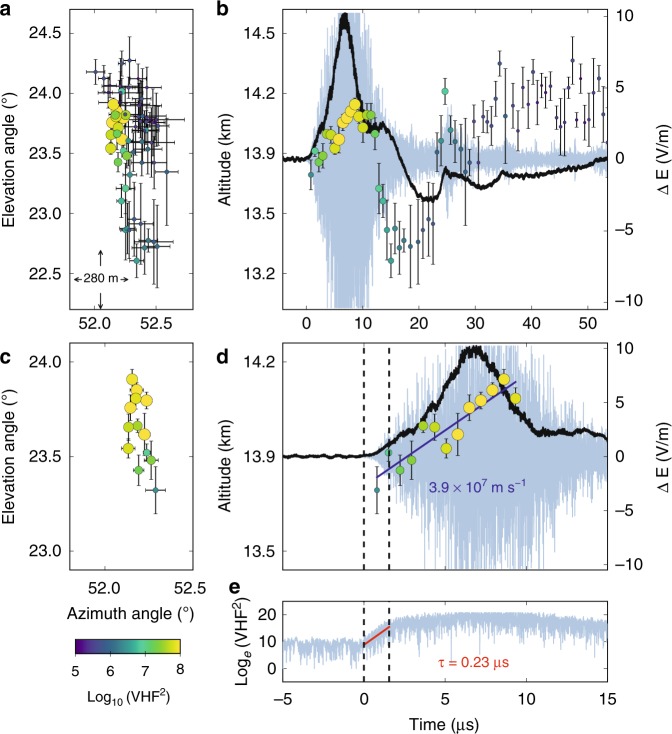
Interferometer data for NBE 9. **a**, **c** Radiation centroids (circular markers, colored and sized by VHF power) for NBE 9, plotted in elevation vs. azimuth, showing the breakdown activity was primarily vertical. Each marker denotes the average angular position of the 128 source solutions in each 0.7-μs window, and error bars denote the standard deviations. **b**, **d** Radiation centroids and fast electric field change (sferic) observations (black waveform) superimposed on the VHF waveform (gray), showing the upward propagation of the VHF source. The positive sferic waveform is indicative of a downward-directed current, consistent with the NBE occurring below the storm’s upper positive charge, and indicating the upward development was due to negative-polarity breakdown. The breakdown ascended ≃400 m in 10 μs, corresponding to a speed of ≃4 × 10^7^ m s^−1^. **e** Semilog plot of the VHF power vs. time, showing the fast exponential rise of the radiation (rise time *τ* = 0.23 μs), coincident with the fast rise of the electric field change (**d**). The peak current of the breakdown was −58 kA

One especially high-rate NBE period (~40 NBEs within 1 min) lasted from 09:58 to 09:59 UT, during which NBEs tended to cluster in space and time (e.g., Stanley et al.^33^). A cluster of ten isolated NBEs that took place between the main negative and upper positive charge regions in the normal-polarity storm^34^ is indicated by a black bracket in Fig. 1b, and is shown expanded in Fig. 1c, where the LMA sources are sized and colored according to source power in dBW. The ten NBEs were preceded and followed by fully developed flashes (not shown), i.e., a negative cloud-to-ground flash ended 3 s before the start of the first NBE in the cluster, and a normal-polarity intra-cloud flash began 0.1 s after the tenth (final) NBE in the cluster. The INTF triggered on the strong VHF radiation from each of the ten NBEs. As discussed in the next section, the INTF and FA data indicate that NBE 1 (Fig. 2) was produced by downward-propagating fast positive breakdown similar to that reported by Rison et al.^8^. In contrast, similarly fast breakdown but upward-directed and of negative polarity produced NBE 2 and NBE 9 (Figs. 3 and 4, respectively). The polarity of the remaining seven NBEs could not be determined because the vertical extent of each event was too small to determine an unambiguous propagation direction.

Despite differences in breakdown polarity and propagation direction, NBEs 1, 2, and 9 are consistent with the high-power NBEs discussed in Rison et al.^8^, having LMA-estimated peak VHF powers of 105, 12, and 43 kW, respectively. Also, the charge-moment changes (−320, −190, and −120 C-m), charge transfers (−0.5, −0.3, and −0.3 C), peak currents (−75, −47, and −58 kA), and current rise *e*-folding times (0.5, 0.4, and 0.4 μs) obtained from simulations of the fast breakdown sferics for NBEs 1, 2, and 9, are similar to the Rison et al.^8^ results. These results are summarized in Table 1.

**Table 1:** Fast breakdown characteristics for NBEs 1, 2, and 9

NBE	Breakdown polarity	*v* (m s^−1^)	Δ*z* (m)	*P*_pk_ (kW)	*I*_pk_ (kA)	*Q*_m_ (C·m)	Δ*Q* (C)	*τ*_1,r_ (μs)	*τ*_1,f_ (μs)	*τ*_2,r_ (μs)	*τ*_2,f_ (μs)
NBE 1	Positive	5.0 × 10^7^	600	105	−75	−320	−0.5	0.5	2.6	1.2	3.7
NBE 2	Negative	4.2 × 10^7^	600	12	−47	−190	−0.3	0.4	3.6	3.9	1.9
NBE 9	Negative	3.9 × 10^7^	400	43	−58	−120	−0.3	0.4	0.4	1.6	2.5

The polarity is that of the initial fast breakdown of each NBE, and does not correspond to the sferic polarity. *v* is the INTF-determined speed of the fast breakdown, Δ*z* is the INTF-determined vertical extent of the fast breakdown, and *P*_pk_ is the LMA-determined peak source power. *I*_pk_ and *Q*_m_ correspond to the peak current and charge-moment change during fast breakdown, as determined from FA sferic simulations similar to those in Rison et al.^8^, and Δ*Q* is the charge transfer. Two double-exponential current pulses were used to simulate each sferic. The first and second current pulses have rise and fall *e*-folding times of *τ*_1,r_, *τ*_1,f_, and *τ*_2,r_, *τ*_2,f_, respectively

### Fast breakdown polarity

The fast electric field change measured by the FA was positive for all three NBEs, indicative of the current being downward directed^35^. The polarity of the events is then determined from INTF observations of the breakdown propagation direction. For each NBE, we construct a series of 0.7 μs-exposure VHF images^36,37^ (also see Methods), and then locate the centroid, or brightest pixel, in each image, to determine the location of the VHF-emitting source over time. To minimize the effects of noise in each 0.7-μs image, which corresponds to 128 VHF samples, we find 128 centroid locations by shifting each imaging window by one sample (5.6 ns) and calculate the average of those 128 locations. The resulting centroid maps for NBEs 1, 2, and 9 are shown in Figs. 2–4, respectively, where the circular markers correspond to the average centroid location in a 0.7-μs time period, and the error bars denote the standard deviation (SD). In particular, for the upward negative-breakdown NBEs, Figs. 3a, c and 4a, c show the centroids of NBE 2 and NBE 9, respectively, in elevation vs. azimuth angle, indicating their development was predominantly vertical, similar to the downward positive-breakdown of NBE 1 in Fig. 2, and in other observations of NBEs^8,38^. Panels b, d of each of the figures show the NBEs’ temporal development, co-plotted with coincident VHF (light blue) and FA (black) waveforms. The altitude vs. time development of the upward negative-breakdown NBEs is similar to that of the downward positive-breakdown example, with both being indicative of a localized source, or single breakdown front (albeit with noticeable scatter in the emitter locations). The measured VHF speeds and rise times are also similar for the three NBEs, ranging from 4 to 5 × 10^7^ m s^−1^ and 0.21–0.24 μs, respectively.

### NBE source characterization

**Fig. 5:**
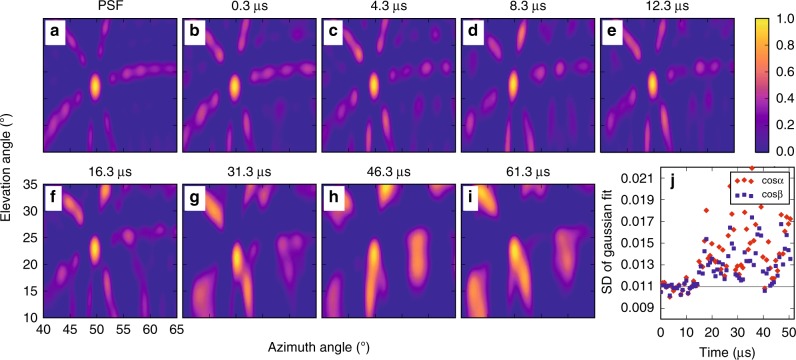
Radio images of NBE 2. Elevation vs. azimuth images of (**a**) an ideal point source at NBE 2’s location (*Az*, $E\ell$ = 50°, 23°), called the point spread function (PSF), **b**–**e** sample images at ≃4 μs intervals during the first 15 μs of NBE 2, corresponding to its upward fast negative breakdown (FNB) phase, and **f**–**i** sample images during successive 15-μs intervals. In each case, the images are 0.7-μs exposures, normalized to the peak amplitude of the centroid. The central lobe of the PSF shows the angular resolution of the three-antenna array and, along with the side lobes, remains essentially unchanged during the FNB, consistent with the VHF radiation being from a localized source. Following the FNB, the central lobe becomes increasingly elongated and the side lobes intensify and become disorganized, indicative of extended and/or multiple radiation sources. **j** Standard deviations (SDs) of the central lobe in the (cos*α*, cos*β*) projection plane vs. time (red, blue symbols), compared to the SDs of the PSF function (gray line), quantitatively showing the radiation to be localized during the FNB and increasingly non-localized and random subsequent to the FNB

Figure 5 shows example 0.7 μs images used in determining the centroid results of the preceding section. The imaging centroid technique differs from that of Rison et al.^8^ in that it makes use of the full cross-correlation functions of antenna pairs, rather than just their peaks^36^. The centroid locations are similar for the two approaches, but the imaging provides additional information about the spatial distribution of the source (see Methods). If the source is localized, i.e., is contained within the angular resolution of the INTF, then its location is well determined by the centroid location. For extended sources, the centroid does not necessarily represent the source location.

As an example, Fig. 5 shows sequential images taken from NBE 2. The intensity distribution in each image is normalized, ranging from 0 (dark blue) to 1 (yellow). For comparison, Fig. 5a shows the simulated image of an ideal (noiseless) point source positioned at azimuth angle (*Az*) and elevation angle $(E\ell )$ of 50° and 23°, respectively, similar to the NBE 2 source locations. The intensity pattern of an ideal point source is called the point spread function (PSF)^37,39^ of the INTF (see Methods), and depends on INTF specifications such as bandwidth, and baseline lengths and orientations. The PSF is characterized by a bright main lobe, with an angular size that indicates the resolution of the INTF, and lower-intensity side lobes. Note that the top row of images (Fig. 5b–e) corresponds to the first 15 μs of NBE 2, and the images are qualitatively similar to the PSF, having a single bright main lobe of comparable size and similarly bright and morphologically similar side lobes. This indicates that the first 15 μs of NBE 2 is consistent with a localized source, and that the sources are well-located by the centroid locations. In contrast, the second row (Fig. 5f–i) corresponds to the latter (≥15 μs) portion of NBE 2, and the images look substantially different from the PSF, having both a broader main lobe and morphologically dissimilar side lobes, as well as higher-intensity content in the side lobes. Such images are inconsistent with a localized source, and the centroid locations may not accurately depict the emitter locations. This is reflected in the increased uncertainty of the altitude values after ~15 μs in Figs. 2–4, and in the increased SDs shown in Fig. 5j.

The VHF images for NBE 2 (Fig. 5b–i) were each fitted with a two-dimensional Gaussian to determine their SDs (see Methods). The resulting values are shown in Fig. 5j, which clearly demonstrates two distinct regimes of activity. During the first 15 μs of NBE 2, i.e., the fast breakdown stage, the SDs in both the cos*α* and cos*β* directions (see Methods for cosine plane projection) are confined to values between 0.010 and 0.012, making the sources consistent with a localized source radiating within the angular resolution of about 1.0° azimuth angle and 3.5° elevation angle. Often, the source is indistinguishable from a point source (with both SDs ≈0.011, corresponding to an angular resolution of 1.6° and 3.8° in azimuth and elevation angle, respectively). In this case, the source locations and progression are well-represented by the centroid values, which show reduced scatter in the propagation (see Figs. 2a, b, 3a, b, and 4a, b). After about 15 μs (Fig. 5f–i), the SDs are noticeably greater and show substantially more scatter in values, ranging between 0.012 and 0.022, indicating the angular source extents are greater than those of the PSF, extending instead up to >1.6° and >5° in azimuth and elevation angle, respectively. In addition, the side lobes are substantially altered. Thus, the latter stage appears to be a mix of localized and extended sources with the centroids not being consistent with a localized source.

**Fig. 6:**
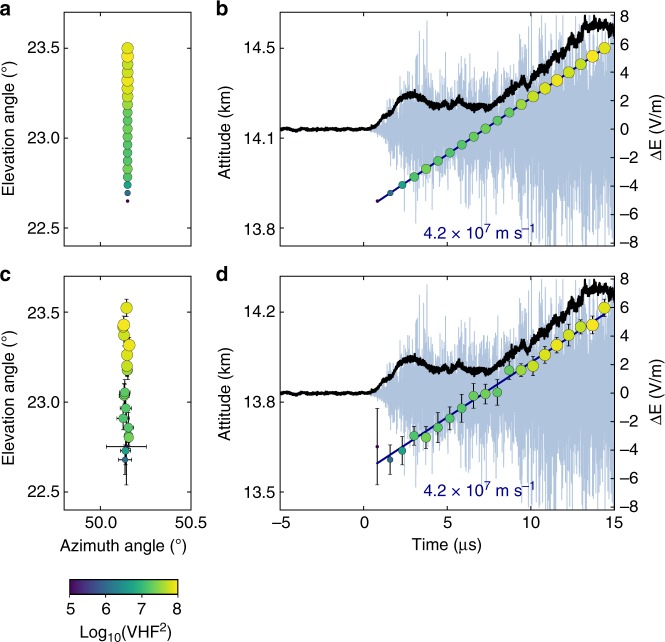
NBE 2 simulation. Simulated radiation centroids (circular markers, colored and sized by VHF power) of a vertically propagating point source, having the same VHF amplitudes, altitudes, and speed of NBE 2. Each marker denotes the average angular position of the 128 source solutions in each 0.7-μs window, and error bars denote the standard deviations. **a**, **b** Centroid locations without added noise, plotted in elevation vs. azimuth and elevation vs. time, respectively, and **c**, **d** same, but with the pre-flash noise of NBE 2 added, showing that the resulting scatter in elevation and azimuth is partially but not fully accounted for by the pre-flash noise

Because the first 15 μs of NBE 2 was consistent with a localized source, four possible models exist to explain the upward-propagating NBE 2 centroid movement depicted in Fig. 3d. The first and simplest model is a point source, or negative breakdown front, moving upward in altitude, which is the model used to obtain Fig. 6 (discussed below). A second plausible model is that of an extended source that grows upward in altitude. In this case, upward negative-polarity breakdown is still required to explain the centroid movement. A third model is that of two point sources, one that moves upward in altitude with greater source power, the other moving downward in altitude with lesser source power, so that the overall altitude change of the centroid is positive. Again, this would require an upward negative-polarity breakdown in order to explain the centroid movement, but would also indicate that a positive breakdown occurs concurrently with fast negative breakdown. Also, the fast negative breakdown speed determined in Fig. 3d would be a lower limit. The fourth model is of two stationary point sources separated by at least 600 m in altitude. If the higher source radiates more strongly over time compared with the lower source, then the centroid movement is an apparent effect. This scenario is not physically plausible. The exponential increase (0.24 μs rise time) of the measured VHF power requires that the VHF power of the higher (more strongly radiating) source should increase on the same timescale. If the lower source does not also increase on the same timescale, then the centroid location is soon determined by the higher source alone (after 1.5 μs, the source power increased by a factor of 500). The upward centroid movement must then be due to upward source propagation, not two stationary sources. Alternatively, if both the higher and lower source powers increase on the same timescale, then it is highly likely that an electrical connection exists between the two sources. This would suggest that an extended emitter (>600 m) already exists at the onset of the exponential rise in VHF power, which is inconsistent with the INTF observations that show that there is no pre-event discharge activity.

Given the above discussion, fast moving, upward breakdown is required to explain the centroid movement of a localized source as depicted in Figs. 3 and 4. Like NBE 2, NBE 9 was also produced by upward fast breakdown of negative polarity, in this case at a speed of 3.9 × 10^7^ m s^−1^ (Fig. 4d). In contrast, the fast breakdown of NBE 1 developed downward, in the same direction as the current, and therefore was of positive polarity (Fig. 2d). In each case, the breakdown was initiated near 13.6 km altitude and had similar vertical extents (400–600 m) and propagation speeds (several times 10^7^ m s^−1^). The features of the initial fast breakdown during NBEs 1, 2, and 9 are summarized in Table 1.

### Fast breakdown simulation

To further validate the breakdown developing as a fast propagating event, we simulate the INTF images of a propagating ideal point source, while taking into account the (pre-flash) noise in the INTF measurement (see Methods). In particular, the initial 15 μs of NBE 2 is modeled as a fast (4.2 × 10^7^ m s^−1^) monotonically ascending point source that begins at 13.6 km altitude and a distance 32.6 km from the INTF, which has the same VHF power per image as for the actual NBE 2 record. Figure 6a, b shows the noise-free model for the fast breakdown of NBE 2, while Fig. 6c, d shows the model with added noise.

Comparison of Figs. 3c, d and 6c, d shows that the scatter in the source locations of the actual measurements is only partially accounted for by the pre-flash noise. Since our simulation of a monotonically ascending point source (Fig. 6c, d) does not fully explain the scatter in centroids that is depicted in the observations (Fig. 3c, d), it is possible that we have either underestimated the noise, that the source speed fluctuates, or that a spatially distributed cascading sequence of activity is responsible for the fast negative breakdown observed for NBE 2 (and NBE 9), and perhaps is also responsible for the fast positive breakdown of NBE 1 and other fast positive breakdown events, given the similarities between fast negative and fast positive breakdown. Regardless, the centroid locations during the initial breakdown of NBE 2 (Fig. 3c, d) are consistent with fast, upward-propagating breakdown that carries a current opposite to its propagation direction, transporting negative charge upward into the upper positive charge region (inset in Fig. 1d), and therefore being of negative polarity.

## Discussion

Though dart leaders^40–42^ and K-processes^43,44^ both constitute negative-polarity breakdown events that can reach speeds in excess of 10^7^ m s^−1^, they occur along a path preconditioned by preceding discharges, whereas fast negative breakdown appears to occur in virgin air. Streamers are the only known form of electrical breakdown in virgin air that can reach speeds on the order of 10^7^ m s^−1^ (refs. ^45–48^), so the high speeds of fast breakdown can be explained by propagating streamers. Although relativistic runaway electron avalanches (RREAs)^49^ also constitute a negative-polarity discharge in virgin air, the propagation speed of the RREAs is 0.89 c^50^, which is much faster than the observed propagation speed of the fast negative breakdown. Because the runaway electron avalanche length at thunderstorm altitudes is approximately a few hundred meters long^51^, the characteristic timescale of an individual RREA is about 1 μs, much shorter than the 10–30-μs duration of the fast negative breakdown. As a result, to create such a long timescale, multiple RREAs would be necessary, but then it is not obvious why these multiple RREAs would cause a discharge to propagate in the direction opposite the electric field direction. Finally, it is not clear how RREAs could produce the large VHF power emitted during the fast negative breakdown, nor do they represent a viable mechanism for producing the observed sferics^52^, given the length scale (<1 km) of fast negative breakdown.

The current magnitude^24,53,54^ and spatial and temporal scales of a single streamer are much smaller than the scales observed during fast breakdown, so a reasonable interpretation of the fast breakdown in NBEs 2 and 9 is that it consists of a system of many negative streamers. The fact that the VHF signal turns on coincidentally with the FA signal and both develop exponentially agrees with the findings of Rison et al.^8^, and is also consistent with modeling of streamer development which shows similar exponential growth^11,24^, regardless of polarity.

Negative streamer development alone in virgin air is not immediately consistent with our understanding of dielectric breakdown in virgin air^20–24,55^. In particular, in investigating the polarity asymmetry in lightning initiation and propagation, it has been suggested that positive streamers originate first at some initiation point and negative streamers are initiated from the point later when the field is sufficiently large^55^. However, negative streamers alone can occur in earth’s atmosphere if positive streamer development is suppressed, for example, at the edge of the ionosphere for negative sprites^48^. In this case, both the nonuniformity of the electric field and the location of the initiating breakdown is critical to the breakdown’s polarity. During the 24 August storm, a similar nonuniformity of the thundercloud electric field is suggested by the high isolated NBE rate, which indicates localized regions of intense electric field^8^. Depending on the location of initiating breakdown relative to the nonuniformity, it is possible that negative streamers could propagate in virgin air for some time without a positive-streamer counterpart.

Fast negative breakdown seems to share many similar characteristics with fast positive breakdown, as is evident from the entries in Table 1. It is interesting to note that the differences between positive and negative streamers cause no significant difference in the characteristics of the two types of fast breakdown. It has been suggested that electron drift motion being convergent for positive breakdown while divergent for negative breakdown leads to more favorable condition for the positive breakdown to start and propagate^55^. The highest electron drift speed in streamers is on the order of 10^5^ m s^−1^, which is two orders of magnitude slower than the fast breakdown propagation speed. With streamer speeds much larger than electron drift speeds, the properties of streamers are dominated by their ionization wave nature. Whether electron drift is in the same direction or opposite direction of the wave then becomes less important, which results in negligible difference between positive and negative streamers^24,56^.

It is interesting to note not only the similarities between the fast negative breakdown events (NBEs 2 and 9) and the fast positive breakdown event (NBE 1) herein, but also the similarities with the three NBE-producing fast positive breakdown events in Rison et al.^8^ (NBEs 1, 2, and 3). The similarities exist despite inherent differences between Florida thunderstorms and New Mexico thunderstorms^34,57–59^, the Florida storms having higher associated MSL heights, higher negative charge centers, and spanning a greater range of temperatures than New Mexico storms, as is also supported by this study. The Rison et al.^8^ NBEs have significantly lower initiation altitudes (9–10 km MSL) compared to those of the NBEs herein (13–14 km MSL). Given that NBE parameters can vary widely, these similarities are surprising, albeit inconclusive, given the small sample of NBEs compared.

Another possibility to consider is that fast negative breakdown is not due to negative streamer development, but is due to positive streamers moving in retrograde motion that gives an apparent upward propagation direction. That is, negative charge deposited at each streamer initiation point^11,54^ could create a sufficiently large electric field to launch positive streamers upwind of the preceding streamers, and so on. Rison et al.^8^ used this idea to explain retrograde development at the beginning of one of their positive-polarity NBEs (NBE 2), which was confirmed by the time-resolved retrograde development of a high-altitude screening discharge. Moreover, such a cascading sequence of activity could explain the scatter discrepancy between the INTF observations and the simulations. However, this still requires a highly localized electric field to limit the overall spatial extent of the fast breakdown in the direction of the thunderstorm field. Furthermore, it is not obvious how a retrograde-motion system of positive streamers can reach 3–5 × 10^7^ m s^−1^ in propagation speed, and so retrograde positive breakdown development appears to be an implausible mechanism for explaining fast negative breakdown.

In summary, high-speed radio interferometric observations show that dielectric breakdown can begin with negative polarity in thunderstorms, which propagates at a speed as high as 4 × 10^7^ m s^−1^, extends over a distance of 500 m, and like other high-power NBEs is a source of the strongest natural VHF emission on Earth. It is unclear what physical mechanism is behind the fast negative breakdown, and a better understanding of the NBE mechanism will have important implications for storm convective strength monitoring^31,32,60,61^ that would be particularly useful for space-borne global monitoring and climatology. The observations thus far show that most NBEs are produced by fast positive breakdown, while those produced by fast negative breakdown are less common but constitute a noticeable portion of events. The properties of the fast negative breakdown suggest it consists of a large number of streamers, whether they are negative streamers propagating opposite the direction of the thunderstorm electric field, positive streamers developing in retrograde motion, or another unforeseen mechanism involving many streamers. It appears, regardless, that a suppression of the normal breakdown development in the thunderstorm electric field direction is required in order for fast negative breakdown to take place without readily discernible processes in the opposite direction.

## Methods

### Interferometer system

**Fig. 7:**
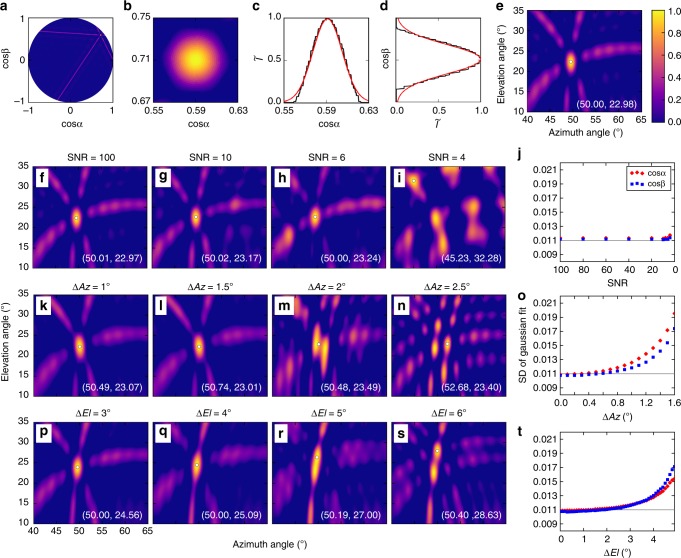
Radio images of different source types. Simulated images and related information of: **a**–**e** an ideal point source, corresponding to the point spread function (PSF) of the antenna array, **f**–**j** the effect of uncorrelated noise on the observations, and the effect of angular separation of two equal-power point sources in azimuth (**k**–**o**), and elevation (**p**–**t**). Each image shows the normalized intensity $(\tilde I)$ of a 0.7-μs exposure of sources at the location of NBE 2. Panels **c** and **d** show that the central lobe of the PSF has a standard deviation (SD) of 0.011 in both the cos*α* and cos*β* directions, corresponding to SDs of 1.6° and 3.8° in azimuth and elevation, respectively. As seen in **j**, added noise has little effect on the size of the central lobe, remaining close to the PSF value of 0.011 (horizontal gray line) for signal-to-noise ratios down to 6. Binary sources have a much stronger effect, with the two sources being readily distinguishable for azimuthal separations of 2° or elevation separations of 5–6°. The fixed central lobe becomes noticeably deformed well before that (**o** and **t**)

The INTF used in this study is a broadband VHF system that employs three antennas operating at 20–80 MHz bandwidth on 100-m equilateral baselines, with automatic triggering of the 180 MHz, 16-bit data recording. The system locates sources in the direction cosine plane, corresponding to the downward projection of the sources from a unit celestial hemisphere onto the plane of the antenna array. The *x* and *y* locations in the cosine plane correspond to the direction cosines (cos*α*, cos*β*) of the source, where *α* is the angle that the source makes with respect to east, and *β* is the angle that the source makes with respect to north. Source locations lie within a circle of unit radius, corresponding to the horizon (Fig. 7a). The values of the direction cosines are determined from the time differences of arrival (TDOAs) at pairs of antennas, obtained by correlating the received signals at pairs of antennas. The TDOAs for the three antenna pairs constrain the source to lie along straight lines in the direction cosine plane, each of which is oriented perpendicular to its respective antenna baseline. The location of the source corresponds to the intersection of the lines. The example shown in Fig. 7a is for a point source at the location of NBE 2. For NBE 2, (cos*α*, cos*β*) = (0.707, 0.592), corresponding to azimuth $\left( {Az = {\mathrm{tan}}^{ - 1}({\mathrm{cos}}\beta /{\mathrm{cos}}\alpha )} \right)$ and elevation $\left( {E\ell = {\mathrm{cos}}^{ - 1}\sqrt {{\mathrm{cos}}^2\alpha + {\mathrm{cos}}^2\beta } } \right)$ values of (*Az*, $E\ell$) = (50°, 23°).

The images are generated based on the innovative algorithm of Stock^36,37^, which uses the full cross-correlation functions to produce the images directly in the cosine plane^36,37^. The size of the central lobe of the image reflects the width of the main cross-correlation peak, with the side lobes corresponding to subsidiary, periodic peaks, oriented according to the baseline directions.

### Image morphologies

The image morphologies are investigated by comparing the observed images to simulated images of known source types. The size of the main lobe is used to quantify the spatial extent of the source. Figure 7 shows simulated images of several source types. The top row of panels corresponds to an ideal point source, the central lobe of which defines the spatial resolution of the array. In this case, the source was positioned at (*Az*, $E\ell$) = (50°, 23°), corresponding to the location of NBE 2. Figure 7a shows the source in the cosine projection plane, and illustrates how the source location is determined, namely from the intersection of lines of constant time difference of arrival at the three pairs of antennas. For the point source, the three peaks in the amplitude vs. time delay of the corresponding cross-correlation functions intersect^36,37^ to create a Gaussian-distributed strong central lobe that is nearly circularly symmetric (Fig. 7b). SD of the lobe is 0.011 in both directions (Fig. 7c, d), namely 1.1% of the overall extent of the cosine projection plane. When projected upward onto the celestial hemisphere to determine the *Az* and $E\ell$ angles, the lobe becomes elongated in elevation, corresponding to SDs of 1.6° in azimuth and 3.8° in elevation (Fig. 7e). The centroid location (white dot) and corresponding azimuth-elevation values (white text) demonstrate that the location of an ideal point source is well determined by the centroid location. The larger scale of the azimuth–elevation plot also shows the characteristic side lobes, which are caused by subsidiary, periodic peaks in the cross-correlation functions. The full image is called the point spread function (PSF) of the array and is a unique function of the antenna geometry^39^, fundamental to interpreting observational data.

Figure 7f–i shows images of the same point source, but with increasing noise (decreasing signal-to-noise ratio, or SNR) in the VHF signal. By simulating a point source at (*Az*, $E\ell$) = (50°, 23°) 100 times with each of the SNR values, we determined that for SNRs >6, on average the centroid is correctly located to within 0.01° in azimuth and elevation angles. Comparing with NBEs 1, 2, and 9, which had SNR values ranging between 10 and 100 and extended about 1° in elevation angle, added noise should have minimal impact on the centroid determinations. An estimate of the INTF noise was obtained from the pre-flash noise levels of the INTF VHF waveforms, determined from the relatively quiet period prior to the NBE in question (for example, the VHF signal (blue waveform) in Fig. 2, prior to time 0 and about 1 ms in duration). The pre-flash noise content was roughly Gaussian, with an SD about 1% of the INTF dynamic range. Added noise minimally affects the image morphology, which is illustrated by comparing the SD of the central lobe with that of the PSF (Fig. 7j), which shows negligible effect down to an SNR of 6.

On the other hand, multiple sources significantly affect the morphology, as illustrated by images of two point sources with increasing angular separation. This is shown in the third and fourth rows of Fig. 7. A single main lobe exists for angular separations less than the angular resolution (1.6° in azimuth and 3.8° in elevation). The main lobe becomes increasingly elongated as the angular separation grows, with the centroid location becoming the average location of the two individual point sources^37^. The two sources become readily distinguishable for an azimuthal separation of 2° or an elevation separation of 5–6°, but elongation of the main lobe becomes noticeable well before that, as seen in the SD comparisons of Fig. 7o, t.

### Angular resolution

As mentioned previously, the INTF PSF in the cosine projection plane (Fig. 7a) has an approximately two-dimensional Gaussian intensity distribution in the main lobe. The resolution in the cosine plane can be approximated from the full-width-half-maximum (FWHM) of the Gaussian fits to the main lobe^37^. The angular resolutions in azimuth and elevation angle as a function of elevation angle $\left( {E\ell } \right)$ is given by ${\mathrm{\Delta }}Az = {\mathrm{FWHM}}/{\mathrm{cos}}(E\ell )$ and ${\mathrm{\Delta }}El = {\mathrm{FWHM}}/{\mathrm{s}}{\mathrm{i}}{\mathrm{n}}(E\ell )$, respectively, similar to Equations (4.4) and (4.5) in Stock^37^. For a source positioned near elevation angle 23°, similar to the NBE sources herein, the angular resolution becomes 1.6° in azimuth angle and 3.8° in elevation angle.

### LMA observations

**Fig. 8:**
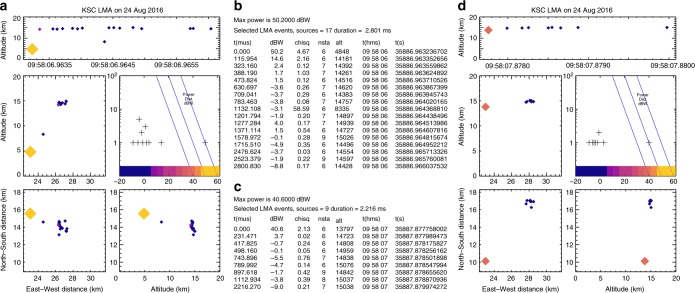
Detailed lightning mapping data for NBEs 1 and 2. **a**, **d** LMA maps of the complete activity for each of the two NBE-initiated discharges, showing how the NBEs are mis-located relative to the subsequent, lower-power activity, and how the subsequent activity more accurately indicates the NBEs actual location. **b**, **c** Numerical listings of the source times, power (in dBW), goodness-of-fit $\chi _\nu ^2$ (chisq) value, and number of stations (nsta) participating in the solutions

Figure 8 shows the detailed KSCLMA observations for NBEs 1 and 2. The sources are sized and colored by the logarithmic source power, with the large yellow/orange sources corresponding to the two NBEs. Despite the relatively high peak power of the NBEs (50.2 and 40.6 dBW, or 105 and 12 kW, respectively), their ensuing breakdown lasted only ≃2–3 ms, and did not initiate full flashes. Also, both NBEs were substantially mis-located in altitude and/or plan location, with their actual locations being indicated by the subsequent lower power activity. Mis-location is a typical feature of high power NBEs, and is due to the VHF radiation being continually noisy around the time of the peak, with different mapping stations detecting slightly different peaks^8^. The mis-locations are consistent with the $\chi _\nu ^2$ goodness-of-fit values being relatively large compared to the subsequent sources, as seen in the numerical listings of Fig. 8. Mis-locations and high $\chi _\nu ^2$ values, coupled with relatively high source powers, turn out to be useful features for identifying fast breakdown, of either polarity.

## Data Availability

The data that support the findings of this study are available through a persistent repository, 10.6084/m9.figshare.7661384.

## Code availability

The code that supports the findings of this study is available from the corresponding authors upon reasonable request.
